# Efficient production of 2′-fucosyllactose from fructose through metabolically engineered recombinant *Escherichia coli*

**DOI:** 10.1186/s12934-024-02312-5

**Published:** 2024-02-01

**Authors:** Ran You, Lei Wang, Meirong Hu, Yong Tao

**Affiliations:** 1 Division of Life Sciences and Medicine, University of Science and Technology of China, Hefei, 230027 China; 2 Chinese Academy of Sciences Key Laboratory of Microbial Physiological and Metabolic Engineering, Institute of Microbiology, Chinese Academy of Sciences, Beijing, 100101 China; 3 Microcyto Biotechnology (Beijing) Co., Ltd., Beijing, 102200 China

**Keywords:** Metabolic engineering, *Escherichia coli*, 2′-Fucosyllactose, High stoichiometric yield, Fed-batch bioconversion

## Abstract

**Background:**

The biosynthesis of human milk oligosaccharides (HMOs) using several microbial systems has garnered considerable interest for their value in pharmaceutics and food industries. 2′-Fucosyllactose (2′-FL), the most abundant oligosaccharide in HMOs, is usually produced using chemical synthesis with a complex and toxic process. Recombinant *E. coli* strains have been constructed by metabolic engineering strategies to produce 2′-FL, but the low stoichiometric yields (2′-FL/glucose or glycerol) are still far from meeting the requirements of industrial production. The sufficient carbon flux for 2′-FL biosynthesis is a major challenge. As such, it is of great significance for the construction of recombinant strains with a high stoichiometric yield.

**Results:**

In the present study, we designed a 2′-FL biosynthesis pathway from fructose with a theoretical stoichiometric yield of 0.5 mol 2′-FL/mol fructose. The biosynthesis of 2′-FL involves five key enzymes: phosphomannomutase (ManB), mannose-1-phosphate guanylytransferase (ManC), GDP-d-mannose 4,6-dehydratase (Gmd), and GDP-l-fucose synthase (WcaG), and α-1,2-fucosyltransferase (FucT). Based on starting strain SG104, we constructed a series of metabolically engineered *E. coli* strains by deleting the key genes *pfkA*, *pfkB* and *pgi*, and replacing the original promoter of *lacY*. The co-expression systems for ManB, ManC, Gmd, WcaG, and FucT were optimized, and nine FucT enzymes were screened to improve the stoichiometric yields of 2′-FL. Furthermore, the gene *gapA* was regulated to further enhance 2′-FL production, and the highest stoichiometric yield (0.498 mol 2′-FL/mol fructose) was achieved by using recombinant strain RFL38 (SG104*ΔpfkAΔpfkBΔpgi119-lacYΔwcaF*::*119-gmd-wcaG-manC-manB*, *119*-AGGAGGAGG-*gapA*, harboring plasmid P30). In the scaled-up reaction, 41.6 g/L (85.2 mM) 2′-FL was produced by a fed-batch bioconversion, corresponding to a stoichiometric yield of 0.482 mol 2′-FL/mol fructose and 0.986 mol 2′-FL/mol lactose.

**Conclusions:**

The biosynthesis of 2′-FL using recombinant *E. coli* from fructose was optimized by metabolic engineering strategies. This is the first time to realize the biological production of 2′-FL production from fructose with high stoichiometric yields. This study also provides an important reference to obtain a suitable distribution of carbon flux between 2′-FL synthesis and glycolysis.

**Supplementary Information:**

The online version contains supplementary material available at 10.1186/s12934-024-02312-5.

## Background

Human milk oligosaccharides (HMOs), the third most abundant solid substance in breast milk, play an indispensable role in the growth and development of newborns [1–3]. HMOs have been confirmed to regulating the intestinal flora, modulate the immune system, and developing the nervous system [4–7]. The biosynthesis of HMOs has become a research hotspot due to its wide application in pharmaceutical and additives [8–12]. 2′-Fucosyllactose (2′-FL), the most abundant component of HMOs, has been approved by the United States Food and Drug Administration (FDA) as Generally Recognized as Safe (GRAS) [13]. The EFSA Panel on Nutrition, Novel Foods and Food Allergens (NDA) has recognized the safety of 2′-FL for infants under the proposed conditions of use [14–16]. 2′-FL has been added to the premium formula and proved to help form the same variety of intestinal flora [17]. Recent researches showed the importance of 2′-FL in reducing neurodegeneration in stroke brain and protecting intestinal epithelial cells against apoptosis [18, 19]. Due to its healthy values and potential medicinal benefits, great attention is focused on 2′-FL in recent years. Microbial synthesis of 2′-FL is regarded as a promising alternative to conventional methods of breast milk extraction, and of chemical synthesis [20, 21]. The de novo synthesis pathway of 2′-FL involved a key precursor, GDP-l-fucose. The biosynthesis of GDP-l-fucose from the precursor fructose-6-phosphate (F-6-P) involves five enzymes, mannose-6-phosphate isomerase (ManA), phosphomannomutase (ManB), mannose-1-phosphate guanylytransferase (ManC), GDP-d-mannose 4,6-dehydratase (Gmd) and GDP-l-fucose synthase (WcaG) [22]. Then 2′-FL is directly synthesized by the readily available precursor lactose and GDP-l-fucose, and the α-1,2-fucosyltransferase (FucT) catalyzes the transfer of a fucosyl residue from GDP-l-fucose to position 2 of the lactose’s galactosyl to obtain 2′-FL [23].

Currently, recombinant host strains such as *Escherichia coli*, *Saccharomyces cerevisiae*, and *Bacillus subtilis* have been constructed to produce 2′-FL. Among these, *E. coli* was used mostly by researchers due to the maturity of genetic tools and the rapidity of culture [11–13, 22, 24, 25]. For example, Huang et al. modularly constructed a recombinant *E. coli* producing 9.12 g/L 2′-FL from glucose and lactose [26]. Moreover, a combinatorial metabolic engineered *E. coli* was constructed by optimizing the expression of key genes and deleting glutathione reductase, and produced 10.3 g/L 2′-FL from glycerol, mannose, fucose and lactose [27]. In addition, Baumgärtner et al. used the λ-Red recombineering technique to integrate 2′-FL biosynthesis genes expression cassettes into chromosome, achieved a plasmid-free *E. coli* strain with a titer of 20.28 g/L 2′-FL from glycerol, fucose and lactose [28]. Furthermore, Chen et al. set a high titer record of 112.5 g/L 2′-FL from glycerol and lactose by using a novel FucT from *Azospirillum lipoferum* [29]. However, all the mentioned studies focused on emphasizing the stoichiometric ratio of 2′-FL to lactose. Meanwhile, the stoichiometric ratios of 2′-FL to precursors of F-6-P were very low or not mentioned, mainly because of the unblocked metabolic flow and the consumption of growth supply during fermentation. The low yields of 2′-FL indicated that only a small amount of precursors F-6-P (form glucose or glycerol) is distributed to 2′-FL production. Therefore, a 2′-FL biosynthesis pathway with high stoichiometric yields in recombinant *E. coli* strains needs to be metabolically optimized. Our laboratory has developed an *E. coli* strain (SG104) in which the glucose utilization system was modified by replacing native *ptsG* (gene ID: 945,651) and *galR* (gene ID: 947,314) with *glk* (gene ID: 946,858) and *zglf* (galactose:H + symporter from *Zymomonas mobilis*) respectively in the chromosome of *E. coli* BW25113, and the accumulation of acetic acid was reduced by deleting *poxB* (gene ID: 946,132) and overexpressing *acs* (gene ID: 948,572) [30]. In the previous study of our laboratory, the efficient production of myo-inositol was accomplished based on the precursor glucose-6-phosphate (G-6-P) by using SG104 as an original strain [31].

In the present study, we aimed to achieve high stoichiometric yields of 2′-FL by guiding the carbon flux of fructose to F-6-P. A series of metabolic engineering strategies were performed in the 2′-FL biosynthesis. First, a biosynthesis pathway for 2′-FL from F-6-P was designed by overexpressing four endogenous enzymes (ManB, ManC, Gmd, and WcaG) and one exogenous enzyme (FucT). Second, *pfkA* (6-phosphofructokinase I, gene ID: 948412), *pfkB* (6-phosphofructokinase II, gene ID: 946230), and *pgi* (G-6-P isomerase, gene ID: 948535) were deleted to enhance metabolic flux to F-6-P, and the promoter of *lacY* (lactose permease, gene ID: 949083) was replaced by 119-promoter to enhance the intake of lactose. Third, the gene *gapA* (gene ID: 947679), encoding glyceraldehyde-3-phosphate dehydrogenase A in glycolysis, was regulated by replacing promoter and inserting rare codons after initiation codon ATG with different numbers. A high stoichiometric yield (0.498 mol 2′-FL/mol fructose) was obtained using strain RFL38 (SG104, *ΔpfkA*, *ΔpfkB*, *Δpgi*, *119-lacY*, *ΔwcaF*::*119-gmd-wcaG-manC-manB*, *119*-AGGAGGAGG-*gapA*, harboring plasmid P30). Finally, the recombinant strain RFL38 was chosen for scaled-up production of 2′-FL, a titer of 41.6 g/L (85.2 mM) was obtained, corresponding to a stoichiometric yield of 0.482 mol 2′-FL/mol fructose and 0.986 mol 2′-FL/mol lactose. It is the first time to realize the production of 2′-FL from fructose in recombinant *E. coli*. This study fills the gap in the research of 2′-FL synthesis in *E. coli* and provides a new efficient approach for 2′-FL bioproduction.

## Results

### Design of 2′-FL biosynthesis pathway from fructose in ***E. coli***

The general pathway for 2′-FL production through the degradation of glucose or glycerol to obtain the key precursor F-6-P is not sufficient due to the low stoichiometric yields [11–13, 24, 27, 29]. Therefore, it is urgently needed to enhance the low stoichiometric yields of 2′-FL from another carbon source. In *E. coli*, fructose was ingested into cells in three routes (Additional file 1: Fig. S1). Route A involves the fructose phosphotransferase system, fructose PTS permease (*fruAB*) ingests and phosphorylates extracellular one mole of fructose to one mole of fructose-1-P (F-1-P) consuming one mole of phosphoenolpyruvate, then 1-phosphofructokinase (*fruK*) phosphorylates F-1-P to fructose-1,6-bisphosphate (F-1,6-2P) flowing to glycolysis. Route B involves PTS membrane-spanning proteins that transport mannose (*manXYZ*), glucitol (*gutA*) and mannitol (*mtlA*). Route C involves a mutant of glucose PTS permease (PtsG-F) and mannose kinase (*mak*) [32].

**Fig. 1:**
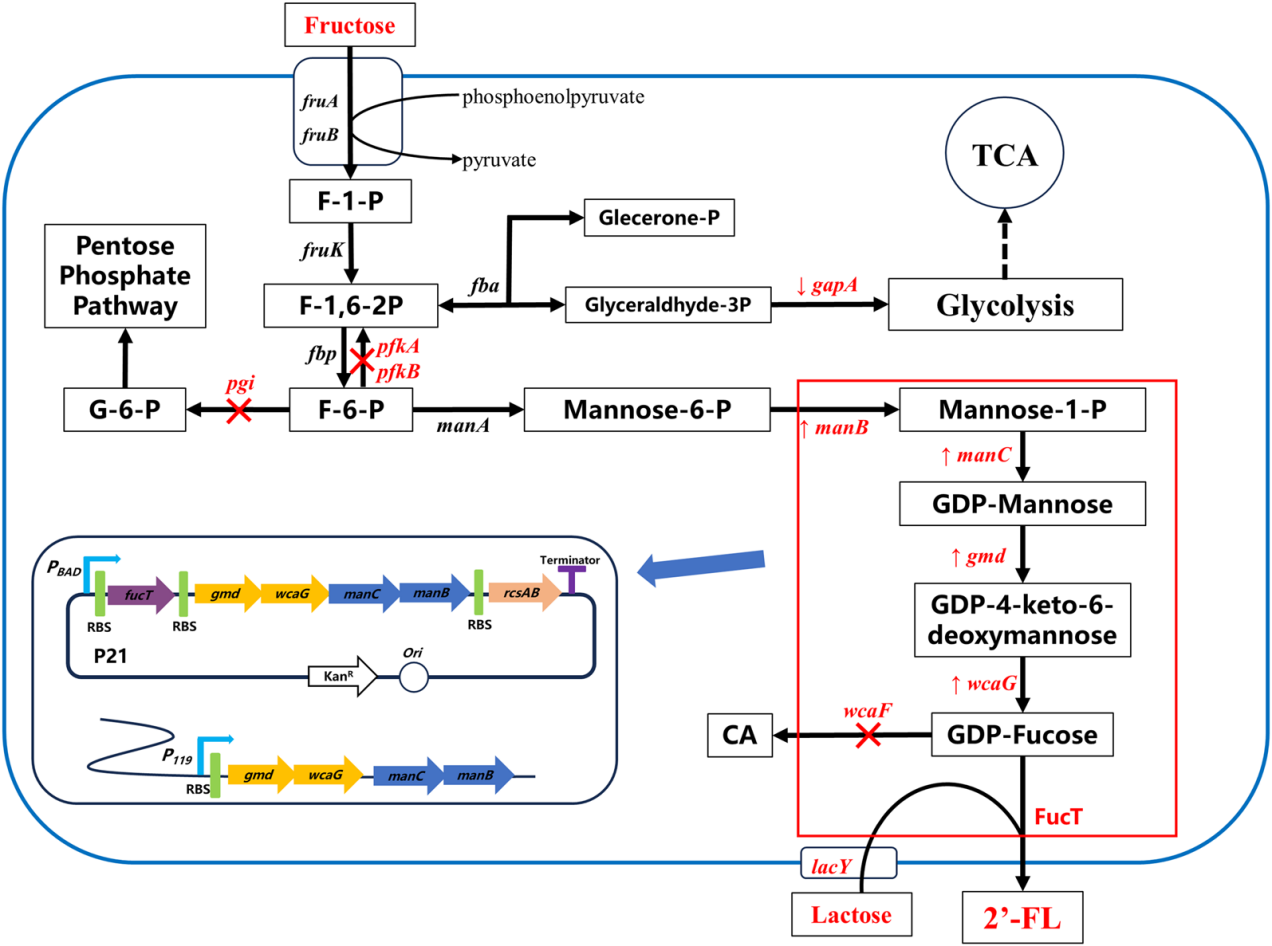
Overview of the 2′-FL biosynthesis pathway in *Escherichia coli*

As such, to bypass the low-yield bottleneck, a biosynthesis pathway of 2′-FL with high stoichiometric yield from fructose was designed by metabolic engineering strategies (Fig. 1). First, fructose is ingested and converted to F-1,6-2P by route A. Second, fructose-1,6-bisphosphase (Fbp) dephosphorylate F-1,6-2P to F-6-P. Third, F-6-P is catalyzed to GDP-mannose by ManA, ManB, and ManC successively. And GDP-mannose is dehydrated by Gmd, then converted to GDP-fucose by WcaG. Finally, a fucosyl residue from GDP-L-fucose is transferred to position 2 of the lactose’s galactosyl by FucT to obtain 2′-FL. In this biosynthetic route under the background of a fructose PTS intake system, the maximum theoretical product yield of 2-FL from d-fructose is 0.5 mol 2′-FL/mol fructose, consuming 1 mol of fructose to generate 0.5 mol of 2-FL.

### Production of 2′-FL by regulating carbon flux in the glycolysis pathway

Controlling carbon flux was crucial for the accumulation of substances of the glycolysis key node [31, 33, 34]. Therefore, the carbon flux was redesigned to improve the stoichiometric yield of 2′-FL. SG104 was chosen as a starting strain that slowed carbon flux to glycolysis to enhance the supply of 2′-FL precursor F-6-P. The key genes involved—*pfkAB* and *pgi*—were respectively deleted. Deletion of *pfkAB* blocks F-6-P flow to glycolysis, and deletion of *pgi* blocks F-6-P flow back to G-6-P. Previous studies have shown that simultaneous overexpression of EcManB, EcManC, EcGmd and EcWcaG from *E. coli* and HpFucT from *Helicobacter Pylori* succeeded in the synthesis of 2′-FL from F-6-P.

**Fig. 2:**
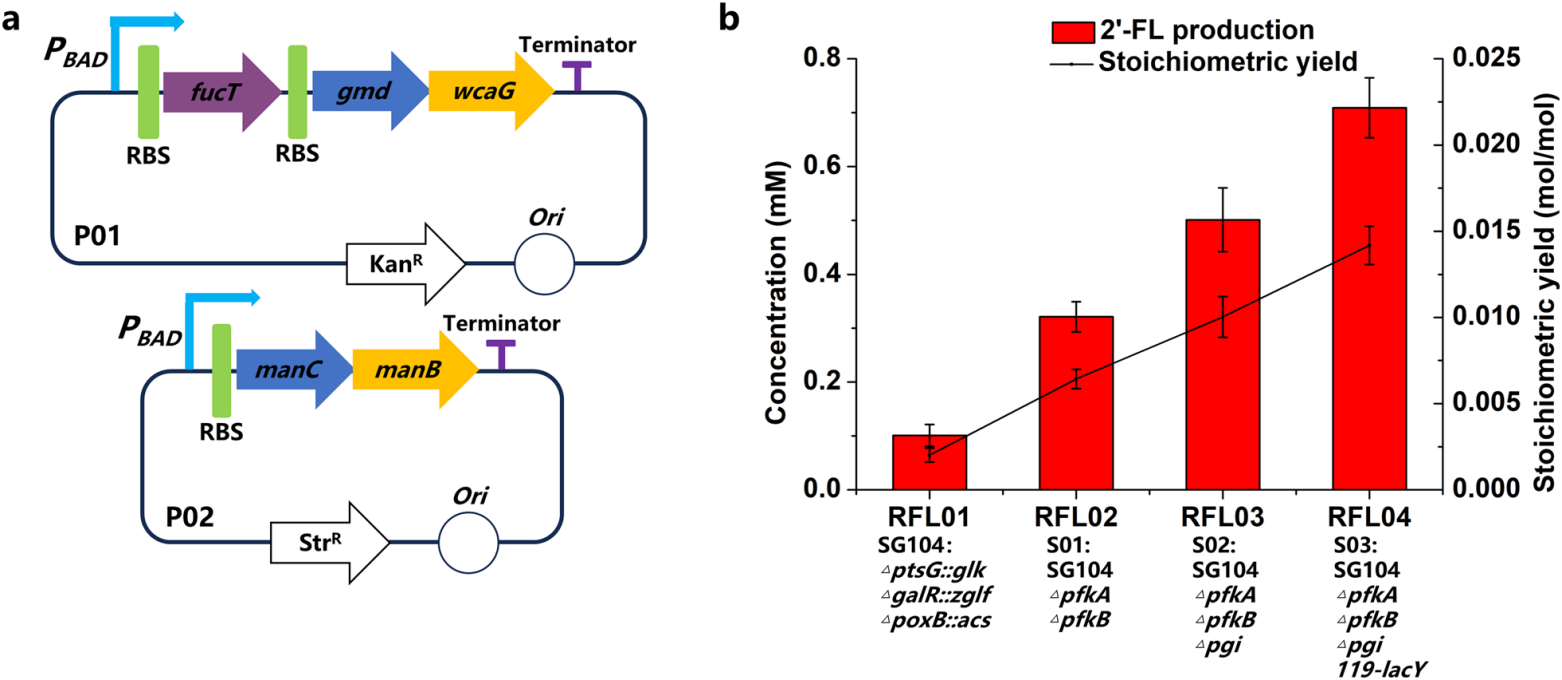
Host strain selection. **a** Schematic diagram of P01 containing the genes encoding FucT, Gmd and WcaG, and P02 containing the genes encoding ManC and ManB. P_BAD_, an araBAD promoter; RBS, ribosome binding sites; Ori, plasmid replication initiation site; Kan^R^, kanamycin resistance gene; Str^R^, streptomycin resistance gene. **b** Production of 2′-FL in different chassis strains. The recombinant strains transformed with plasmids P01 and P02 were induced and then suspended in a bioconversion mixture containing 50 mM fructose and 50 mM lactose. The bioconversions were performed for 8 h at 37 ℃ and 220 rpm

Plasmid P01 expressing HpFucT-EcGmd-EcWcaG and P02 expressing EcManC-EcManB (Fig. 2a) were co-transformed into *E. coli* strains SG104, S01, S02 and S03 respectively to construct recombinant strains RFL01, RFL02, RFL03 and RFL04 for 2′-FL production. As shown in Fig. 2b, 0.71 mM 2′-FL was obtained using RFL04, and the stoichiometric yield reached 0.014 mol 2′-FL/mol fructose. Compared with strain RFL01, strain RFL03 in which *pfkAB* and *pgi* are deleted showed increased 2′-FL production. The results showed that deletions of *pfkAB* and *pgi* were effective for accumulation of precursor F-6-P. Strains RFL04 showed higher 2′-FL titer than RFL03 because of enhancing lactose transport by the substitution of strong promoter (*119-lacY*). As such, strain RFL04 was used in follow-up experiments.

## Enhancement of 2′-FL production by optimizing plasmid expression systems

**Fig. 3:**
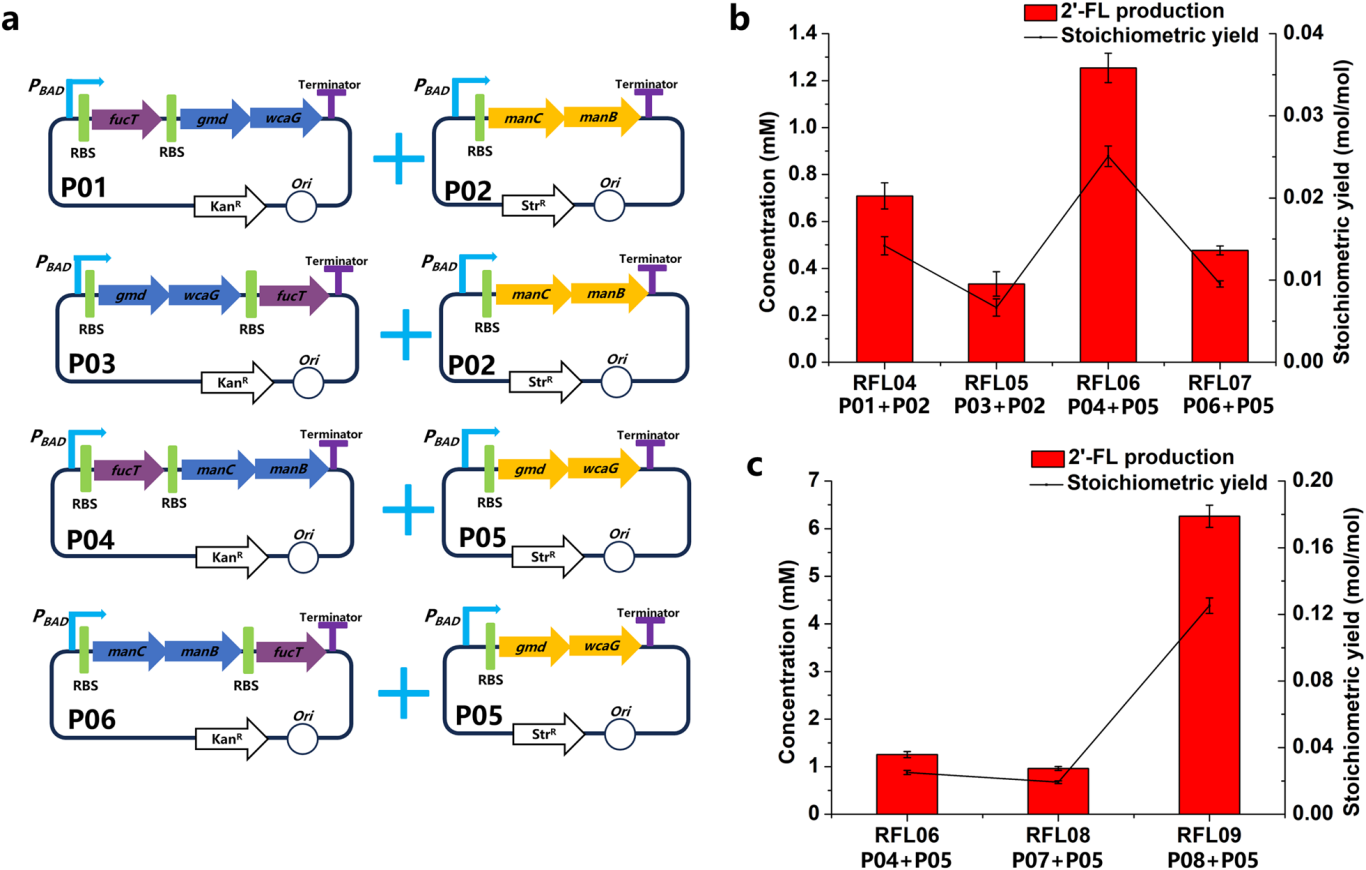
Effects of different plasmid combinations in host strain S03. **a** Schematic diagram of P01 + P02, P03 + P02, P04 + P05, and P06 + P05 combinations. **b** 2′-FL production using different plasmid combinations of **a**. **c** Effects of FucT expression by changing copy number of P04

Due to the low stoichiometric yield of RFL04, further optimization was designed to enhance the production of 2′-FL. Plasmid expression systems are useful for the reconstruction of biosynthesis pathways and usually give a high yield of a target product. The combination of two plasmids with different replicons was used to overexpress enzymes which could catalyze F-6-P to 2′-FL through five-step bioreactions (Fig. 3a). The key exogenous gene *fucT* encoding FucT was constructed at different locations of high copy number plasmids, and the endogenous genes *gmd-wcaG* and *manC-manB* were respectively constructed close to *fucT* or at another medium–low copy number plasmid. For that, plasmids P03, P04, P05 and P06 were constructed. Strain S03 was cotransformed different combination of above high and medium–low copy number plasmids to construct recombinant strains RFL05, RFL06 and RFL07. The expression of synthesis pathway enzymes is shown in Additional file 1: Fig. S2. Among them, RFL06 (S03 harboring P04 + P05) showed a high titer of 1.3 mM and stoichiometric yield of 0.025 mol 2′-FL/mol fructose (Fig. 3b).

**Fig. 4:**
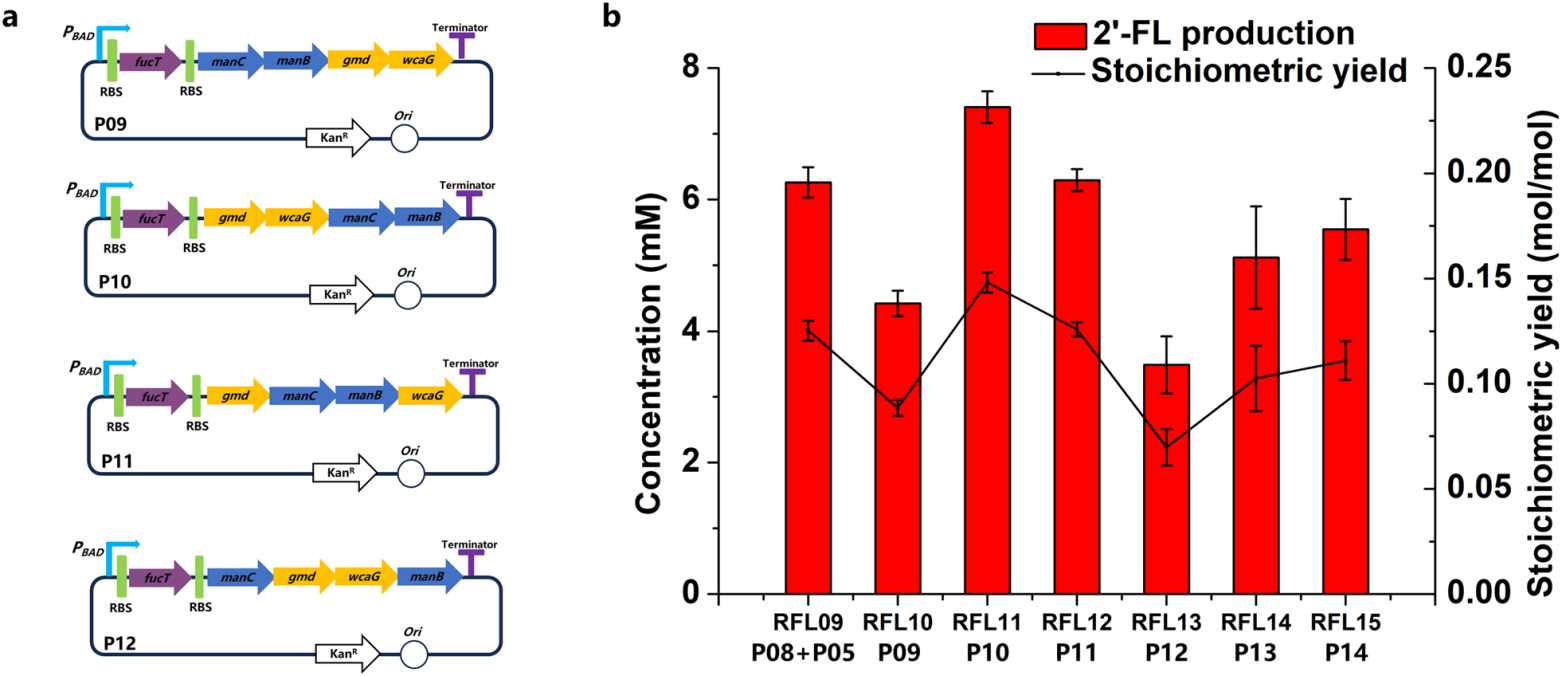
Effects of different marshalling sequences of five genes in integrated plasmids in host strain S03. **a** Schematic diagram of P09–P012 plasmids. **b** 2′-FL production using different integrated plasmids. The recombinant strains RFL09–RFL15 were induced and then suspended in a bioconversion mixture containing 50 mM fructose and 50 mM lactose. The bioconversions were performed for 8 h at 37 ℃ and 220 rpm

GDP-fucose and lactose were synthesized to 2′-FL by FucT which was reported as one of the rate-limiting step in 2′-FL biosynthesis [27, 28, 35]. Therefore, the activity of FucT is one of the most important factors. Plasmids P07 and P08 (not shown in figure) with lower copy numbers than P04 were constructed to increase soluble expression of FucT. Plasmid combinations P07 + P05, and P08 + P05 were respectively transformed into host strain S03 to produce 2′-FL. RFL09 (S03 harboring P08 + P05) produced 0.125 mol 2′-FL/mol fructose with a titer of 6.3 mM. To provide space for another plasmid and achieve stable expression of synthesis pathway enzymes for post-industrial scale-up, the above plasmid combinations were blended into one plasmid expressing five enzymes. Plasmids P09, P10, P11, and P12 were constructed and transformed into host strain S03 to produce 2′-FL. RFL11 (S03 harboring P10) exhibited 2′-FL production of 7.4 mM, corresponding to a stoichiometric yield of 0.148 mol 2′-FL/mol fructose (Fig. 4b). Afterwards, plasmids P13 and P14 (not shown in the figure) were constructed to verify whether the copy number of P10 could reduce. However, RFL14 (S03 harboring P13) and RFL15 (S03 harboring P14) both showed lower titer than RFL11 (Fig. 4b). Comparison with 0.014 mol 2′-FL/mol fructose of RFL04, the stoichiometric yield of RFL11 was improved to 0.148 mol 2′-FL/mol fructose.

### Improvement of endogenous synthesis pathway in chromosome and identification of rate-limiting step

**Fig. 5:**
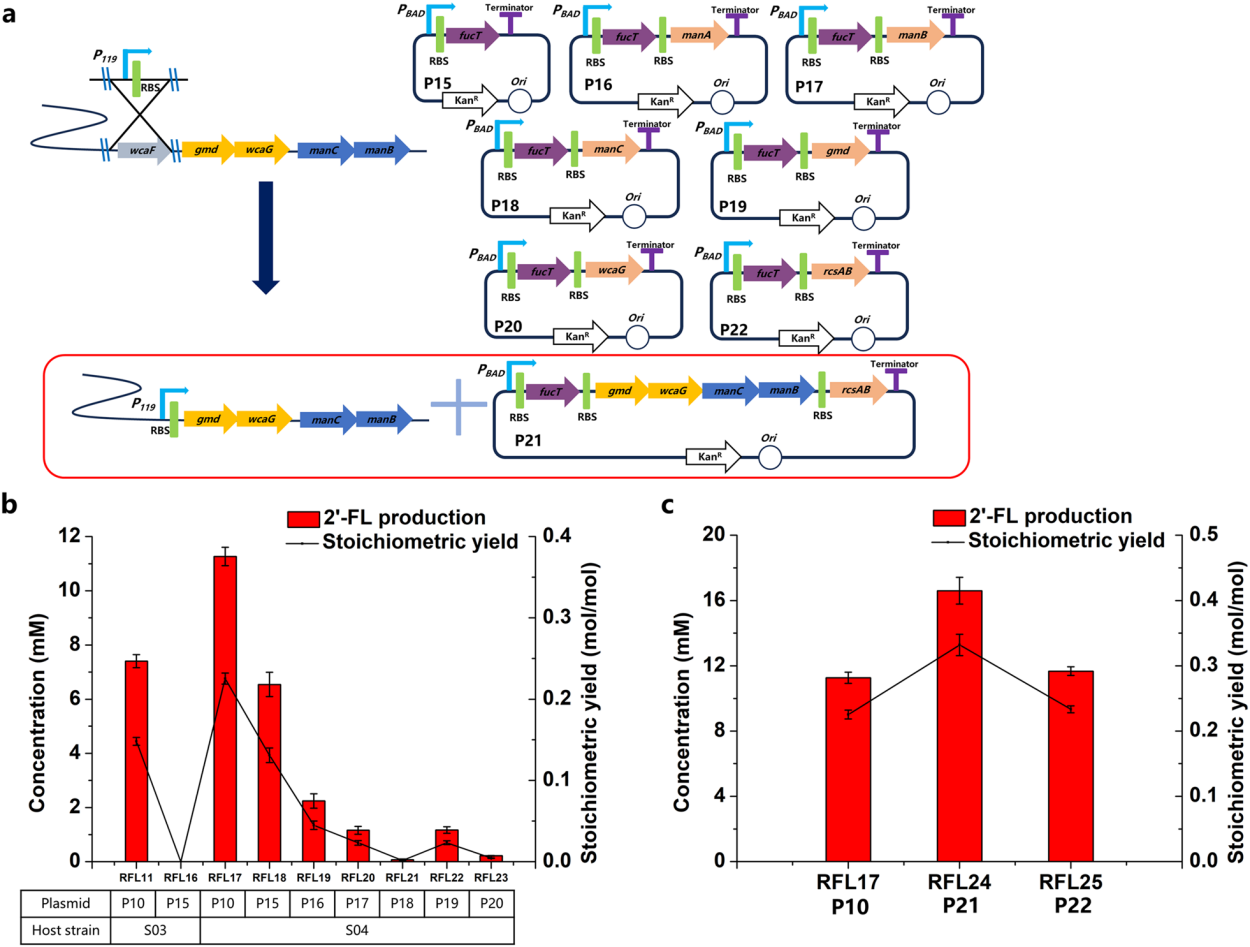
Further enhancement of 2′-FL biosynthesis pathway combining chromosome and plasmid. **a** Schematic diagram of enhancing endogenous enzymes in chromosome and plasmids. P_119_, a constitutive strong promoter. **b** Identification of rate-limiting step for 2′-FL production using different plasmids with chassis strains S03 and S04. **c** Effect of co-overexpression of *gmd-wcaG-manC-manB-rcsAB* cluster in plasmids and *gmd-wcaG-manC-manB* cluster in chromosome for 2′-FL production

To further optimize the GDP-fucose bioconversion from F-6-P, and identify the rate-limiting steps of four endogenous enzymes, *wcaF* encoding colanic acid acetyltransferase was deleted and replaced with strong promoter P119 (Fig. 5a). *wcaF* is involved in by-product colanic acid biosynthesis, and is located in front of *gmd-wcaG-manC-manB* cluster in the chromosome (Fig. 5a). Accordingly, plasmid P15 was transformed into the host strain S04 to verify the importance of plasmid endogenous genes. Recombinant strains RFL16 (S03 harboring P15), RFL17 (S04 harboring P10) and RFL18 (S04 harboring P15) were constructed. Among them, RFL17 exhibited a titer of 11.3 mM and a stoichiometric yield of 0.225 mol 2′-FL/mol fructose (Fig. 5b).

To identify which endogenous enzyme is the rate-limiting step, plasmid P16, P17, P18, P19, and P20 were respectively transformed into S04, and strains RFL19, RFL20, RFL21, RFL22, and RFL23 were constructed. As shown in Fig. 5b, RFL18 showed a higher titer than RFL19 to 23. This indicated that overexpressing of single endogenous enzyme in plasmids could disrupt the relatively stable strength of the endogenous enzymes, after overexpressing *gmd-wcaG-manC-manB* cluster in the chromosome. RcsAB, a DNA-binding transcriptional dual regulator, has been confirmed to up-regulate endogenous enzymes intensity at the same time. Plasmid P21 and P22 were constructed and transformed, then strains RFL24 and RFL25 were used to compare the effects of different expressions of plasmids with RcsAB. RFL25 came to a similar titer with RFL17, indicating that *gmd-wcaG-manC-manB* cluster or *rcsAB* in plasmids enhanced the endogenous biosynthesis pathway to a similar level. And 0.332 mol 2′-FL/mol fructose was produced with a titer of 16.6 mM by RFL24, higher than RFL17 and RFL25 (Fig. 5c). The result suggests that the co-overexpression of *gmd-wcaG-manC-manB-rcsAB* cluster in plasmids and *gmd-wcaG-manC-manB* cluster in the chromosome brings the synthetic pathway to a higher intensity.

### Further screening α1,2-fucosyltransferase to improve 2′-FL production

**Fig. 6:**
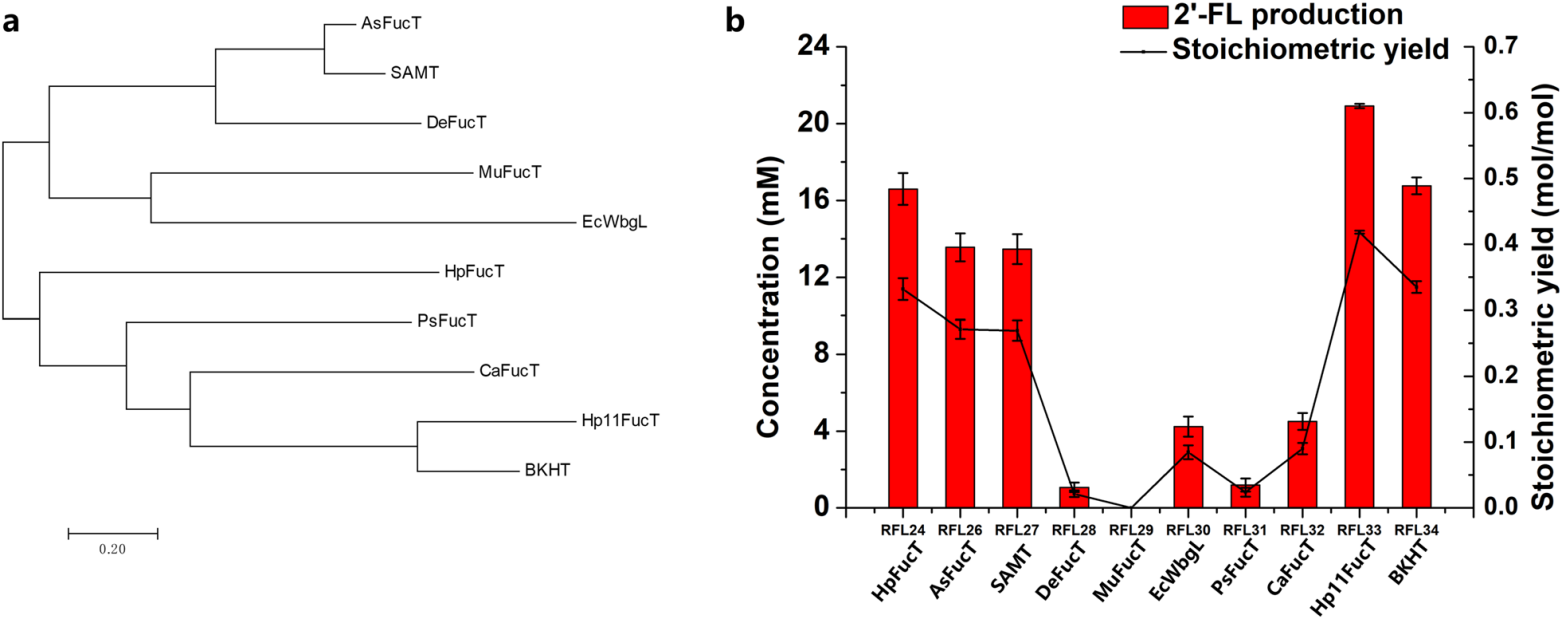
Screen of α1,2-fucosyltransferase to improve 2′-FL production. **a** An evolutionary tree of ten FucT enzymes. **b** Effects of replacement of FucT in plasmid P21 with chassis host S04. The recombinant strains RFL24, RFL26–RFL34 were induced and then suspended in a bioconversion mixture containing 50 mM fructose and 50 mM lactose, and the bioconversions were performed for 8 h at 37 ℃ and 220 rpm

According to previous studies, FucT (α1,2-fucosyltransferase) is responsible for directly synthesizing 2′-FL from GDP-fucose and lactose in the last step. Nine different FucT genes were compared with HpFucT in producing 2′-FL. HpFucT gene of plasmid P21 were replaced respectively by AsFucT from *Azospirillum *sp*.*, SAMT [29], DeFucT from *Deltaproteobacteria bacterium*, MuFucT from *Muribaculaceae bacterium*, EcWbgL from *E. coli*, PsFucT from *Prevotella *sp*.*, CaFucT from *Candidatus Bathyarchaeota archaeon*, Hp11FucT from *Helicobacter *sp*. 11S02629-2* and BKHT [35] genes. The evolutionary relationships of HpFucT and above nine FucT enzymes were exhibited as a maximum likelihood tree analyzed by software MEGA7 (Fig. 6a). As shown in Fig. 6b, 20.9 mM 2′-FL was obtained using RFL33 (S04 harboring P30) expressing Hp11FucT and a stoichiometric yield of 0.418 mol 2′-FL/mol fructose was reached. After a screen of ten FucT enzymes, Hp11FucT from *Helicobacter *sp*. 11S02629-2* showed the highest activity for 2′-FL biosynthesis and was used to produce 2′-FL in subsequent works.

### Strains optimization by regulating *gapA*

**Fig. 7:**
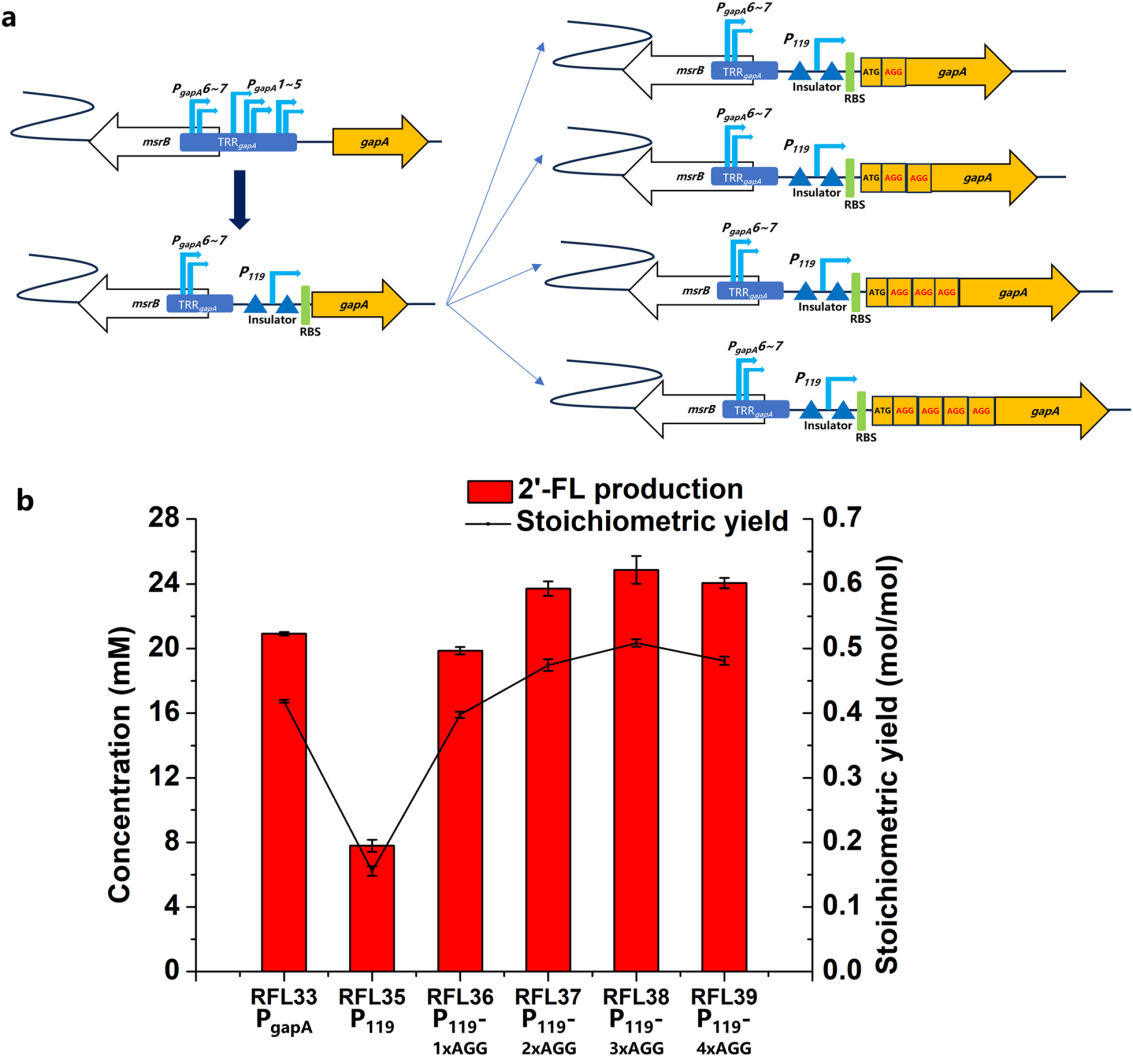
Effects of regulating *gapA* on 2′-FL production. **a** Schematic diagram of replacing the original promoter and inserting rare codons after initiation codon of *gapA*. **b** Production of 2′-FL in different host strains. The recombinant strains were induced and harvested, then suspended in a bioconversion mixture containing 50 mM fructose and 50 mM lactose. The bioconversions were performed at 37 ℃ and 220 rpm for 8 h

The above work achieved a stoichiometric yield of 0.418 mol 2′-FL/mol fructose, not even close to the theoretical conversion yield. Therefore, metabolic regulation was used to optimize host strains for reaching the theoretical stoichiometric ratio. Although degradation pathway of F-6-P has been blocked, the consumption of F-1,6-2P still needs to solved. The gene *gapA,* encoding glyceraldehyde-3-phosphate dehydrogenase (GapA), is necessary for cell growth and acts in the glycolysis reaction of glyceraldehyde-3-phosphate to 3-phospho-glyceroyl phosphate. Therefore, the strength of expression of gene *gapA* was adjusted by replacement of its promoters and insertion of *E. coli* rare codon AGG of different numbers after initiation codon ATG (Fig. 7a). In *E. coli* genome, seven promoters of *gapA* gene work together to regulate its transcription, two of which overlap with the upstream *msrB* gene. Following the principle of not affecting other genes, five *gapA* promoters not overlapping with *msrB* gene were replaced by 119-promoter with insulators both front and rear firstly. Strain S04 was chosen as the platform strain to construct four host strains (S05 to S09), which were used to evaluate the effects of enhancing and blocking GapA to varying degrees.

The resulting host strains were then transformed with the plasmid P30, and strains RFL35 to RFL39 were constructed. Strain RFL38 replacing original promoter with 119-promoter and inserting AGGAGGAGG after initiation codon ATG, exhibited 2′-FL production of 24.9 mM after 8 h of bioconversion, corresponding to a stoichiometric yield of 0.498 mol 2′-FL/mol fructose (Fig. 7b), close to the theoretical conversion rate. In a word, our series of work including adjusting carbon flux, optimizing plasmid expression systems, screening FucT and regulating *gapA*, were proved to be effective. To comprehensively evaluate the overall production performance of 2’-FL, scaled-up production using strain RFL38 was carried out in a 1-L fed-batch fermenter. After 48 h of bioconversion, 85.2 mM 2′-FL was obtained, corresponding to a titer of 41.6 g/L, while 176.9 mM fructose and 86.4 mM lactose were consumed. The stoichiometric yield was 0.482 mol 2′-FL/mol fructose and 0.986 mol 2′-FL/mol lactose (Additional file 1: Fig. S3).

## Discussion

2′-FL and other HMOs are the main functional substances of human milk, displaying a variety of biological activities and pharmaceutical values [1–6]. In recent years, the metabolic engineering and synthetic biology tools have been combined to design microorganisms with desirable functions. *S. cerevisiae*, *Pichia pastoris*, *B. subtilis*, and *E. coli* have been constructed by metabolic engineering strategies to produce 2′-FL using glucose, glycerol, fucose, or sucrose as the carbon sources, but the stoichiometric yields were lower than 0.2 mol/mol [10, 22, 27, 36–38]. *E. coli* is considered a “generally regarded as safe” organism, and metabolic engineering of *E. coli* can efficiently supply F-6-P to enhance the production of 2′-FL. Current studies have explored a variety of approaches for obtaining 2′-FL with high concentrations using glycerol or glucose as carbon sources of in *E. coli* [11–13, 24, 35, 39, 40]. For efficient accumulation of F-6-P, fructose is a potential carbon source. As shown in Additional file 1: Fig S1, fructose intake involves three routes in *E. coli* [32]. Fructose PTS system of route A (Additional file 1: Fig S1) was considered to play an absolute role in the biosynthetic pathway. Meanwhile, *manXYZ* and *mak* genes of route B and C (Additional file 1: Fig S1) had been enhanced by the replacement of strong promoter, but achieved no observable effect (data not shown). In our study, the key targets *pfkA*, *pfkB*, and *pgi* were deleted to enhance the supply of F-6-P and the transport protein LacY was overexpressed to ensure enough intracellular lactose supply (Fig. 1).

In this study, we found that the accumulation of F-6-P was beneficial for the production of 2′-FL (Fig. 2). This was substantiated by the increase in stoichiometric yield observed upon knockout of *pfkA*, *pfkB*, and *pgi*. The activities of five enzymes catalyzing precursor F-6-P to 2′-FL in the biosynthesis pathway are important to the effective synthesis of 2′-FL. Increasing the activities of enzymes ManB, ManC, Gmd, WcaG and FucT by optimizing plasmid co-overexpression systems of *fucT*, *gmd-wcaG-manC-manB* cluster and *rcsAB* in plasmids, and upregulation of *gmd-wcaG-manC-manB* cluster in chromosome exhibited great potential for boosting the production of 2′-FL (Fig. 5). Previous studies found that FucT is the crucial enzyme for directly for converting GDP-fucose and lactose to 2′-FL in the last step [29, 35, 41]. HpFucT and nine other FucT were analyzed through an evolutionary tree of MEGA7 software (Fig. 6a), and constructed into plasmid P21 to screen the enzyme with the best effects of 2′-FL production (Fig. 6b).

Previous experiences have indicated that weakening or deletion of the branching pathway is effective in increasing the yield of the target product [31, 33, 34]. Fructose-bisphosphate aldolase was downregulated by weakening *fbaA* and deleting *fbaB*, but achieving no increase in stoichiometric yield (not shown in data). Thereafter, the key gene *gapA* in the glycolysis was modified by replacing its promoter and inserting different numbers of *E. coli* rare codon AGG to further accumulate F-6-P. In the present 2′-FL biosynthesis pathway, 1 mol of fructose is, in principle, metabolized to 0.5 mol of 2′-FL via a nine-step reaction under the background of a PTS-fructose intake system. The highest yield in our study was 0.498 mol 2′-FL/mol fructose in strain RFL38, approaching the theoretical stoichiometric yield (Fig. 7b). In scaled-up bioconversion of strain RFL38, 41.6 g/L (85.2 mM) 2′-FL was produced, corresponding to a stoichiometric yield of 0.482 mol 2′-FL/mol fructose and 0.986 mol 2′-FL/mol lactose. The strategy is expected to achieve industrial production of 2′-FL at low cost.

Through the above different strategies of metabolic engineering and molecular biological regulations, recombinant *E. coli* with a yield close to the theoretical stoichiometric ratio (0.5 mol 2′-FL/mol fructose) was realized. Besides the PTS fructose intake pathway dependent on PEP, another fructose permease independent on PEP, and fructose phosphorylase with high enzyme activities should be considered for screening. These strategies increase the theoretical conversion rate, expected to further reduce production costs.

## Conclusions

In the present study, we used different engineering strategies to construct *E. coli* recombinant strains producing 2′-FL. Glycolysis in *E. coli* was blocked by inactivating *pfkA*, *pfkB*, and *pgi* genes to redirect the carbon flux. Then the plasmid co-expression systems of five enzymes were optimized to enhance metabolic flux to 2′-FL biosynthesis. Thereafter, screening the crucial enzyme FucT of the last step further increased the stoichiometric ratio. Finally, gene *gapA* necessary for cell growth was appropriately downregulated to further block carbon flux to glycolysis and TCA. The combination of these approaches increased the stoichiometric yield successively to 0.014, 0.225, 0.332, 0.418, and 0.498 mol 2′-FL/mol fructose. Therefore, these strategies of designing engineered strains and performing bioconversion in shake flask and fermenter may be promising for 2′-FL industrial production, and also valuable for the biosynthesis of other compounds with glycolysis node substances as precursors.

### Strains, plasmids and reagents

T4 DNA ligase and restriction enzymes were purchased from New England Biolabs (USA). Gibson kits (2.5 × OK Clon Master Mix) were purchased from Accurate Biotechnology (Hunan) Co., Ltd. (Changsha, China). Plasmid extraction and gel purification kits were purchased from Omega (Beijing, China). DNA polymerase (I-5^™^ 2 × High-Fidelity Master Mix, TP001) was bought from Beijing Tsingke Biotech Co., Ltd. Media components were bought from Becton–Dickinson (Beijing, China). Standards of fructose, lactose, 2′-FL, and other chemicals were obtained from Sigma-Aldrich (Shanghai, China). All *E. coli* strains were grown in Luria–Bertani (LB) medium containing 10 g/L tryptone, 10 g/L NaCl, and 5 g/L yeast extract.

The bacteria strains and plasmids used in this study are listed in Table 1. The plasmids pYB1k, pLB1s, pRB1k, pXB1k, pLB1k, and pSB1k used for the expression of genes are derived from our laboratory’s vectors which have the origin of replication, kanamycin, and streptomycin resistance genes, an araBAD promoter (pBAD), multiple cloning sites and a rrnB terminator. The FucT gene from *Helicobacter Pylori* is codon-optimized and synthesized by GenScript Co., Ltd. (Jiangsu, China). The encoding nucleotide sequence of the enzymes used for producing 2′-FL were amplified by PCR reactions and ligated into the vectors between *Nco*I and *EcoR*I sites by T4 ligation and Gibson assembly method [42]. *E. coli* transT1 was used for molecular cloning. *E. coli* BW25113 was used as the parental cell for genetic modification and 2′-FL production. Gene knock-out strains were obtained according to the KEIO collection (National BioResource Project) [43, 44]. The P1 virus-mediated transfection was used to integrate phenotype of the chromosome [45, 46]. The Crispr–Cas9 system was used for gene knockout, gene replacement and change of ribosomal binding site or promoter [47]. The primers used in the study are listed in Additional file 1: Table S1.

**Table 1:** Strains and plasmids used in this study

Plasmid/strain	Relevant characteristics	Reference
*Plasmids*		
pYB1k	p15A ori, pBAD promoter, Kan^R^	Laboratory
pLB1s	R6K ori, pBAD promoter, Str^R^	Laboratory
pRB1k	RSF1030, pBAD promoter, Kan^R^	Laboratory
pXB1k	p15A ori, pBAD promoter, Kan^R^	Laboratory
pLB1k	R6K ori, pBAD promoter, Kan^R^	Laboratory
pSB1k	pSC101 ori, pBAD promoter, Kan^R^	Laboratory
P01	pYB1k containing HpFucT-EcGmd-EcWcaG genes	Laboratory
P02	pLB1s containing EcManC-EcManB genes	Laboratory
P03	pYB1k containing EcGmd-EcWcaG-HpFucT genes	Laboratory
P04	pYB1k containing HpFucT-EcManC-EcManB genes	This study
P05	pLB1s containing EcGmd-EcWcaG genes	This study
P06	pYB1k containing EcManC-EcManB-HpFucT genes	This study
P07	pRB1k containing HpFucT-EcManC-EcManB genes	This study
P08	pXB1k containing HpFucT-EcManC-EcManB genes	This study
P09	pXB1k containing HpFucT-EcManC-EcManB-EcGmd-EcWcaG genes	This study
P10	pXB1k containing HpFucT-EcGmd-EcWcaG-EcManC-EcManB genes	This study
P11	pXB1k containing HpFucT-EcManC-EcGmd-EcWcaG-EcManB genes	This study
P12	pXB1k containing HpFucT-EcGmd-EcManC-EcManB-EcWcaG genes	This study
P13	pLB1k containing HpFucT-EcGmd-EcWcaG-EcManC-EcManB genes	This study
P14	pSB1k containing HpFucT-EcGmd-EcWcaG-EcManC-EcManB genes	This study
P15	pXB1k containing HpFucT gene	This study
P16	pXB1k containing HpFucT-EcManA genes	This study
P17	pXB1k containing HpFucT-EcManB genes	This study
P18	pXB1k containing HpFucT-EcManC genes	This study
P19	pXB1k containing HpFucT-EcGmd genes	This study
P20	pXB1k containing HpFucT-EcWcaG genes	This study
P21	pXB1k containing HpFucT-EcGmd-EcWcaG-EcManC-EcManB-RcsAB genes	This study
P22	pXB1k containing HpFucT-RcsAB genes	This study
P23	pXB1k containing AsFucT-EcGmd-EcWcaG-EcManC-EcManB-RcsAB genes	This study
P24	pXB1k containing SAMT-EcGmd-EcWcaG-EcManC-EcManB-RcsAB genes	This study
P25	pXB1k containing DeFucT-EcGmd-EcWcaG-EcManC-EcManB-RcsAB genes	This study
P26	pXB1k containing MuFucT-EcGmd-EcWcaG-EcManC-EcManB-RcsAB genes	This study
P27	pXB1k containing EcWbgL-EcGmd-EcWcaG-EcManC-EcManB-RcsAB genes	This study
P28	pXB1k containing PsFucT-EcGmd-EcWcaG-EcManC-EcManB-RcsAB genes	This study
P29	pXB1k containing CaFucT-EcGmd-EcWcaG-EcManC-EcManB-RcsAB genes	This study
P30	pXB1k containing Hp11FucT-EcGmd-EcWcaG-EcManC-EcManB-RcsAB genes	This study
P31	pXB1k containing BKHT-EcGmd-EcWcaG-EcManC-EcManB-rcsAB genes	This study
*Strains*		
*E. coli* trans T1	Wild-type	Invitrogen
*E. coli* BW25113	*lacI*^*q*^*rrnB*_*T14*_∆*lacZ*_*WJ16*_*hsdR514*∆*araBAD*_*AH33*_∆*rhaBAD*_*LD78*_	Invitrogen
SG104	*E. coli* BW25113,*ΔptsG::glk,ΔgalR::zglf,ΔpoxB::acs*	Laboratory
S01	SG104,*ΔpfkA,ΔpfkB*	This study
S02	SG104,*ΔpfkA,ΔpfkB,Δpgi*	This study
S03	SG104,*ΔpfkA,ΔpfkB,Δpgi*,*119-lacY*	This study
S04	S03,*ΔwcaF*::*119-gmd-wcaG-manC-manB*	This study
S05	S04,*119-gapA*	This study
S06	S04,*119-*AGG*-gapA*	This study
S07	S04,*119-*AGGAGG*-gapA*	This study
S08	S04,*119-*AGGAGGAGG*-gapA*	This study
S09	S04,*119-*AGGAGGAGGAGG*-gapA*	This study
RFL01	SG104 harboring plasmids P01 and P02	This study
RFL02	S01 harboring plasmids P01 and P02	This study
RFL03	S02 harboring plasmids P01 and P02	This study
RFL04	S03 harboring plasmids P01 and P02	This study
RFL05	S03 harboring plasmids P03 and P02	This study
RFL06	S03 harboring plasmids P04 and P05	This study
RFL07	S03 harboring plasmids P06 and P05	This study
RFL08	S03 harboring plasmids P07 and P05	This study
RFL09	S03 harboring plasmids P08 and P05	This study
RFL10	S03 harboring plasmid P09	This study
RFL11	S03 harboring plasmid P10	This study
RFL12	S03 harboring plasmid P11	This study
RFL13	S03 harboring plasmid P12	This study
RFL14	S03 harboring plasmids P13	This study
RFL15	S03 harboring plasmids P14	This study
RFL16	S03 harboring plasmid P15	This study
RFL17	S04 harboring plasmid P10	This study
RFL18	S04 harboring plasmid P15	This study
RFL19	S04 harboring plasmid P16	This study
RFL20	S04 harboring plasmid P17	This study
RFL21	S04 harboring plasmid P18	This study
RFL22	S04 harboring plasmid P19	This study
RFL23	S04 harboring plasmid P20	This study
RFL24	S04 harboring plasmid P21	This study
RFL25	S04 harboring plasmid P22	This study
RFL26	S04 harboring plasmid P23	This study
RFL27	S04 harboring plasmid P24	This study
RFL28	S04 harboring plasmid P25	This study
RFL29	S04 harboring plasmid P26	This study
RFL30	S04 harboring plasmid P27	This study
RFL31	S04 harboring plasmid P28	This study
RFL32	S04 harboring plasmid P29	This study
RFL33	S04 harboring plasmid P30	This study
RFL34	S04 harboring plasmid P31	This study
RFL35	S05 harboring plasmid P30	This study
RFL36	S06 harboring plasmid P30	This study
RFL37	S07 harboring plasmid P30	This study
RFL38	S08 harboring plasmid P30	This study
RFL39	S09 harboring plasmid P30	This study

### Culture and bioconversion conditions

LB medium was used for all molecular construction experiments and strain cultures. For expression of proteins, the strains were cultured in LB medium with opportune antibiotics (kanamycin or streptomycin 50 mg/L) at 37 °C and 220 rpm. The strains were incubated at 25 °C and 220 rpm by adding appropriate l-arabinose (0.2 g/L).

The recombinant strains were induced and harvested, then suspended in a bioconversion mixture containing 50 mM fructose and 50 mM lactose. The bioconversions were performed for 8 h at 37 °C and 220 rpm in 1 × M9 salt buffer (Na_2_HPO_4_·7H_2_O 12.8 g/L, KH_2_PO_4_ 3 g/L, NaCl 0.5 g/L, NH_4_Cl 1 g/L) containing 2 mM MgSO_4_. For the scale-up production of 2′-FL, the bioconversions were used for 2′-FL production with the recombinant strain RFL38. The induced cells were harvested by centrifugation and suspended in 500 mL buffer containing 1 × M9 salt with 2 mM MgSO_4_ in a 1-L fermenter. The fructose was added by fed-batch. Cells were cultured with 30–50% oxygen dissolved at 37 °C with a biomass of OD_600_ = 20.

### Analytical methods

Cell density was estimated by measuring the optical density at 600 nm with a spectrophotometer. Recombinant enzyme expression was compared and analyzed by SDS-PAGE. For the preparation of SDS-PAGE samples, induced cells were harvested and suspended in 50 mM phosphate buffer (pH = 7.0) with a cell density of OD_600_ = 10. The cells were lysis by ultrasonic disruption. The mixture was centrifuged, and the supernatant was mixed with an isometric 2 × protein loading buffer. After boiling for 10 min, equal volumes of sample were loaded onto gels. Concentrations of glucose, fructose, lactose, and 2′-FL in the supernatant were measured by HPLC with a Bio-Rad Aminex HPX-87 H column (7.8 × 300 mm; Hercules, CA, USA), refractive index detector (RID). Samples taken from bioconversions were centrifuged, and HPLC samples were obtained by filtration–sterilization of the supernatants. The analysis was performed with a flow rate of 0.5 mL/min using 5 mM H_2_SO_4_ as the mobile phase at 50 °C. The retention times of 2′-FL, fructose and lactose were 8.421 min, 9.109 min, and 11.715 min respectively (Additional file 1: Fig. S4).

### Supplementary Information

**Additional file 1: **Supplementary materials.

## Abbreviations

OD_600_: The optical density of cells at 600 nm
FucT: α-1,2-Fucosyltransferase
*E. coli*: *Escherichia coli*
F-6-P: Fructose-6-phosphate
TCA: Tricarboxylic acid cycle
RBS: Ribosome binding site
pBAD: An araBAD promoter
SDS-PAGE: Sodium dodecyl sulfonate-polyacrylamide gel electrophoresis
HPLC: High performance liquid chromatography
RID: Refractive index detector

## References

[1] Wallingford JC, Myers PN, Barber CM (2022). Effects of addition of 2-fucosyllactose to infant formula on growth and specific pathways of utilization by in healthy term infants. Front Nutr.

[2] Vandenplas Y, Berger B, Carnielli VP, Ksiazyk J, Lagström H, Luna MS, Migacheva N, Mosselmans JM, Picaud JC, Possner M (2018). Human milk oligosaccharides: 2-Fucosyllactose (2-FL) and Lacto-N-Neotetraose (LNnT) in infant formula. Nutrients.

[3] Alliet P, Vandenplas Y, Roggero P, Jespers SNJ, Peeters S, Stalens JP, Kortman GAM, Amico M, Berger B, Sprenger N (2022). Safety and efficacy of a probiotic-containing infant formula supplemented with 2′-Fucosyllactose: a double-blind randomized controlled trial. Nutr J.

[4] Vazquez E, Barranco A, Ramirez M, Gruart A, Delgado-Garcia JM, Jimenez ML, Buck R, Rueda R (2016). Dietary 2′-Fucosyllactose enhances operant conditioning and long-term potentiation via gut–brain communication through the vagus nerve in rodents. Plos ONE.

[5] Van den Abbeele P, Sprenger N, Ghyselinck J, Marsaux B, Marzorati M, Rochat F (2021). A comparison of the in vitro effects of 2′Fucosyllactose and lactose on the composition and activity of gut microbiota from infants and toddlers. Nutrients.

[6] Salli K, Anglenius H, Hiryonen J, Hibberd AA, Ahonen I, Saarinen MT, Tiihonen K, Maukonen J, Ouwehand AC (2019). The effect of 2′-fucosyllactose on simulated infant gut microbiome and metabolites; a pilot study in comparison to GOS and lactose. Sci Rep.

[7] Li AL, Li Y, Zhang X, Zhang CW, Li TT, Zhang JJ, Li C (2021). The human milk oligosaccharide 2′-fucosyllactose attenuates β-lactoglobulin-induced food allergy through the miR-146a-mediated toll-like receptor 4/nuclear factor-kB signaling pathway. J Dairy Sci.

[8] Zhou WT, Jiang H, Liang XX, Qiu YJ, Wang LL, Mao XZ (2022). Discovery and characterization of a novel α-l-fucosidase from the marine-derived and its application in 2′-fucosyllactose production. Food Chem.

[9] Zhang ZY, Li YT, Wu MJQ, Gao Z, Wu B, He BF (2023). Identification and Characterization of a novel a-l-fucosidase from enterococcus gallinarum and Its application for production of 2′-Fucosyllactose. Int J Mol Sci.

[10] Wan L, Zhu YY, Chen G, Luo GC, Zhang WL, Mu WM (2021). Efficient production of 2′-Fucosyllactose from l-Fucose self-assembling multienzyme complexes in engineered *Escherichia coli*. ACS Synth Biol.

[11] Zhang QW, Liu ZM, Xia HZ, Huang ZY, Zhu YL, Xu LF, Liu YF, Li JH, Du GC, Lv XQ, Liu L (2022). Engineered *Escherichia coli* for the de novo production of 2′-fucosyllactose. Microb Cell Fact.

[12] Ni ZJ, Li ZK, Wu JY, Ge YF, Liao YX, Yuan LX, Chen XS, Yao JM (2020). Multi-path optimization for efficient production of 2′-Fucosyllactose in an engineered *Escherichia coli* C41 (DE3) derivative. Front Bioeng Biotechnol.

[13] Liu YL, Zhu YY, Wan L, Chen RL, Zhang WL, Mu WM (2022). High-level biosynthesis of 2′-Fucosyllactose by metabolically engineered *Escherichia coli*. J Agric Food Chem.

[14] Turck D, Bohn T, Castenmiller J, De Henauw S, Hirsch-Ernst KI, Maciuk A, Mangelsdorf I, McArdle HJ, Naska A, Pelaez C (2022). Safety of the extension of use of 2′-fucosyllactose/difucosyllactose (2′-FL/DFL) mixture and lacto-tetraose (LNT) as novel foods in food supplements for infants pursuant to Regulation (EU) 2015/2283. EFSA J.

[15] Turck D, Castenmiller J, De Henauw S, Hirsch-Ernst KI, Kearney J, Maciuk A, Mangelsdorf I, McArdle HJ, Naska A, Pelaez C (2019). Safety of 2′-fucosyllactose/difucosyllactose mixture as a novel food pursuant to Regulation (EU) 2015/2283. EFSA J.

[16] Turck D, Bohn T, Castenmiller J, De Henauw S, Hirsch-Ernst KI, Maciuk A, Mangelsdorf I, McArdle HJ, Naska A, Pelaez C (2022). Safety of 2′-Fucosyllactose (2'-FL) produced by a derivative strain (APC199) of corynebacterium glutamicum ATCC 13032 as a novel food pursuant to Regulation (EU) 2015/2283. EFSA J.

[17] Zhu YY, Wan L, Li W, Ni DW, Zhang WL, Yan X, Mu WM (2022). Recent advances on 2′-Fucosyllactose: physiological properties, applications, and production approaches. Crit Rev Food Sci Nutr.

[18] Wu KJ, Chen YH, Bae EK, Song Y, Min W, Yu SJ (2020). Human milk oligosaccharide 2′-Fucosyllactose reduces neurodegeneration in stroke brain. Transl Stroke Res.

[19] Zhao G, Williams J, Washington MK, Yang YH, Long JR, Townsend SD, Yan F (2022). 2′-Fucosyllactose ameliorates chemotherapy-induced intestinal mucositis by protecting intestinal epithelial cells against apoptosis. Cell Mol Gastroenterol Hepatol.

[20] Pereira CL, McDonald FE (2012). Synthesis of human milk oligosaccharides: 2′- and 3′-Fucosyllactose. Heterocycles.

[21] Agoston K, Hederos MJ, Bajza I, Dekany G (2019). Kilogram scale chemical synthesis of 2′-Fucosyllactose. Carbohyd Res.

[22] Parschat K, Schreiber S, Wartenberg D, Engels B, Jennewein S (2020). High-titer biosynthesis of the predominant human milk oligosaccharide 2′-Fucosyllactose from sucrose in *Escherichia coli*. ACS Synth Biol.

[23] Li C, Wu M, Gao X, Zhu ZL, Li Y, Lu FP, Qin HM (2020). Efficient biosynthesis of 2′-Fucosyllactose using an in vitro multienzyme cascade. J Agric Food Chem.

[24] Li ML, Luo YJ, Hu MM, Li CC, Liu Z, Zhang T (2022). Module-guided metabolic rewiring for fucosyllactose biosynthesis in engineered *Escherichia coli* with lactose de novo pathway. J Agric Food Chem.

[25] Li ML, Li CC, Hu MM, Zhang T (2022). Metabolic engineering strategies of de novo pathway for enhancing 2 '-Fucosyllactose synthesis in *Escherichia coli*. Microb Biotechnol.

[26] Huang D, Yang KX, Liu J, Xu YY, Wang YY, Wang R, Liu B, Feng L (2017). Metabolic engineering of *Escherichia coli* for the production of 2′-fucosyllactose and 3-fucosyllactose through modular pathway enhancement. Metab Eng.

[27] Lin L, Gong MY, Liu YF, Li JH, Lv XQ, Du GC, Liu L (2022). Combinatorial metabolic engineering of *Escherichia coli* for de novo production of 2′-fucosyllactose. Bioresour Technol.

[28] Baumgärtner F, Seitz L, Sprenger GA, Albermann C (2013). Construction of *Escherichia coli* strains with chromosomally integrated expression cassettes for the synthesis of 2′-fucosyllactose. Microb Cell Fact.

[29] Chen Y, Zhu Y, Wang H, Chen R, Liu Y, Zhang W, Mu W (2023). De novo biosynthesis of 2′-fucosyllactose in a metabolically engineered *Escherichia coli* using a novel a1,2-fucosyltransferase from Azospirillum lipoferum. Bioresour Technol.

[30] Zhang S, Yang W, Chen H, Liu B, Lin B, Tao Y (2019). Metabolic engineering for efficient supply of acetyl-CoA from different carbon sources in *Escherichia coli*. Microb Cell Fact.

[31] You R, Wang L, Shi CR, Chen H, Zhang SS, Hu MR, Tao Y (2020). Efficient production of myo-inositol in *Escherichia coli* through metabolic engineering. Microb Cell Fact.

[32] Kornberg HL (2001). Routes for fructose utilization by *Escherichia coli*. J Mol Microbiol Biotechnol.

[33] Brockman IM, Prather KLJ (2015). Dynamic knockdown of *E. coli* central metabolism for redirecting fluxes of primary metabolites. Metab Eng.

[34] Tang EJ, Shen XL, Wang J, Sun XX, Yuan QP (2020). Synergetic utilization of glucose and glycerol for efficient myo-inositol biosynthesis. Biotechnol Bioeng.

[35] Zhu YY, Chen RL, Wang H, Chen YH, Liu YL, Zhou JW, Mu WM (2023). Elimination of byproduct generation and enhancement of 2′- Fucosyllactose synthesis by expressing a novel α1,2-Fucosyltransferase in engineered. J Agric Food Chem.

[36] Xu MY, Sun MT, Meng XF, Zhang WX, Shen Y, Liu WF (2023). Engineering pheromone-mediated quorum sensing with enhanced response output increases fucosyllactose production in saccharomyces cerevisiae. ACS Synth Biol.

[37] Qian DF, Zhang CY, Deng C, Zhou M, Fan LQ, Zhao LM (2023). De novo biosynthesis of 2′-fucosyllactose in engineered *Pichia pastoris*. Biotech Lett.

[38] Ji MH, Liu YF, Xie SQ, Fu C, Liu M, Shi JP, Sun JS (2022). De novo synthesis of 2'-fucosyllactose in engineered *Bacillus subtilis* ATCC 6051a. Process Biochem.

[39] Li ML, Li CC, Luo YJ, Hu MM, Liu Z, Zhang T (2022). Multi-level metabolic engineering of *Escherichia coli* for high-titer biosynthesis of 2′-fucosyllactose and 3-fucosyllactose. Microb Biotechnol.

[40] Li CC, Li ML, Hu MM, Zhang T (2023). Metabolic engineering of de novo pathway for the production of 2′-fucosyllactose. Mol Biotechnol.

[41] Engels L, Elling L (2014). WbgL: a novel bacterial α1,2-fucosyltransferase for the synthesis of 2'-fucosyllactose. Glycobiology.

[42] Gibson DG, Young L, Chuang RY, Venter JC, Hutchison CA, Smith HO (2009). Enzymatic assembly of DNA molecules up to several hundred kilobases. Nat Methods.

[43] Yamamoto N, Nakahigashi K, Nakamichi T, Yoshino M, Takai Y, Touda Y, Furubayashi A, Kinjyo S, Dose H, Hasegawa M (2009). Update on the Keio collection of *Escherichia coli* single-gene deletion mutants. Mol Syst Biol.

[44] Baba T, Ara T, Hasegawa M, Takai Y, Okumura Y, Baba M, Datsenko KA, Tomita M, Wanner BL, Mori H (2006). Construction of *Escherichia coli* K-12 in-frame, single-gene knockout mutants: the Keio collection. Mol Syst Biol.

[45] Thomason LC, Costantino N, Court DL (2007). *E coli* genome manipulation by P1 transduction. Curr Protoc Mol Biol.

[46] Datsenko KA, Wanner BL (2000). One-step inactivation of chromosomal genes in *Escherichia coli* K-12 using PCR products. Proc Natl Acad Sci U S A.

[47] Jiang Y, Chen B, Duan C, Sun B, Yang J, Yang S (2015). Multigene editing in the *Escherichia coli* genome via the CRISPR–Cas9 system. Appl Environ Microbiol.

