# Supplementary material for: Efficient production of 2′-fucosyllactose from fructose through metabolically engineered recombinant Escherichia coli
Source: Microb Cell Fact. 2024 Feb 1;23:38. doi: 10.1186/s12934-024-02312-5 (PMC10835893; doi:10.1186/s12934-024-02312-5)
Supplement: Supplementary file 1 — Additional file 1: Supplementary materials. [file 12934_2024_2312_MOESM1_ESM.docx]

**Efficient production of 2’-fucosyllactose from fructose through metabolically engineered recombinant *Escherichia coli***

Ran You^1,2^ Lei Wang^2,3*^ Meirong Hu^2^ and Yong Tao ^2,3*^

^1^ Division of Life Sciences and Medicine, University of Science and Technology of China, Hefei 230027, China.

^2^ Chinese Academy of Sciences Key Laboratory of Microbial Physiological and Metabolic Engineering, Institute of Microbiology, Chinese Academy of Sciences, Beijing 100101, China.

^3^ Microcyto Biotechnology (Beijing) Co.,Ltd. Beijing 102200, China.

*Correspondence: wl8893@163.com.com; taoyong@im.ac.cn

**Additional file 1**

**Table S1** Primers used in this study.

| Primers | Sequence (5’-3’) |
| --- | --- |
| **2’FL-synthetic pathway** |  |
| EcManB-F | atgaaaaaattaacctgctt |
| EcManB-R | ttactcgttcagcaacgtca |
| EcManC-F | atggcgcagtcgaaactctatc |
| EcManC-R | ttacacccgtccgtagcgat |
| EcGmd-F | atgtcaaaagtcgctctcat |
| EcGmd-R | ttatgactccagcgcgatcg |
| EcWcaG-F | atgagtaaacaacgagtttt |
| EcWcaG-R | ttacccccgaaagcggtcttg |
| HpFucT-F | ATGGCTTTTAAAGTAGTTCA |
| HpFucT-R | TTAGGCGTTATATTTCTGGC |
| AsFucT-F | ATGATTATAGTAAGGTTAAC |
| AsFucT-R | TTAGCCGTCCAAGCGCGTCC |
| SAMT-F | ATGATTATAGTAAGGCTATC |
| SAMT-R | TTAGCCGTCGAGGCGAAGCC |
| DeFucT-F | ATGGCTCCACTACCCCGAGA |
| DeFucT-R | TTACAAACGAACCCAACGAT |
| MuFucT-F | ATGAAAATAGTAAATATTAC |
| MuFucT-R | TTACTTCTTCAGCTCCAACG |
| EcWbgL-F | ATGAGTATTATAAGGTTACA |
| EcWbgL-R | ttaGCAACTGGAATGTTTAT |
| PsFucT-F | ATGGTAACAGTTTTGTTATC |
| PsFucT-R | TTAAATCTTCTTCCAGCCAT |
| CaFucT-F | ATGGGAAATCAGATTTTTCA |
| CaFucT-R | TTAATAGCTGATTTTTACCC |
| Hp11FucT-F | ATGATTGAAATAAGGTTACA |
| Hp11FucT-R | TTAGCGCAGGATCAGCTTCT |
| BKHT-F | ATGAAAAATAAAGTACAGAT |
| BKHT-R | TTATTGGCCATAACGAAAGG |
| RcsA-F | atgtcaacgattattatgga |
| RcsA-R | ttagcgcatgttgacaaaaatac |
| RcsB-F | atgaacaatatgaacgtaat |
| RcsB-R | ttagtctttatctgccggac |
| **gene deletion** |  |
| *Δpgi*-up-F | cattttcagccttggcacaa |
| *Δpgi*-up-R | GGCCTACATATCGACGATGATAGCAATACTCTTCTGATTT |
| *Δpgi*-down-F | aaatcagaagagtattgctatcatcgtcgatatgtaggcc |
| *Δpgi*-down-R | GGCAAAAATGCCATACAGAAC |
| *ΔpfkA*-up-F | TATATAGCGCGTTACGCATG |
| *ΔpfkA*-up-R | TCCGAAATCAGACTACCTCTGAACTTTGGAATGCAAAATG |
| *ΔpfkA*-down-F | AGAGGTAGTCTGATTTCGGAAAAAGGCAGATTCCTTTACC |
| *ΔpfkA*-down-R | GTGACTGACGAATCACCACG |
| *ΔpfkB*-up-F | cagtggtgtgtccataccag |
| *ΔpfkB*-up-R | atgctgggggaatgtttttgcatttcctcctataggctga |
| *ΔpfkB*-down-F | tcagcctataggaggaaatgcaaaaacattcccccagcat |
| *ΔpfkB*-down-R | cctgccacatgatgtctctc |
| **promoter replacement** |  |
| *lacY*-up-F | ttgatggtagtggtcaaatg |
| 119*lacY*-up-R | acgacgtggtgttagctgtgttatttttgacaccagacca |
| 119*lacY*-mid-F | tggtctggtgtcaaaaataacacagctaacaccacgtcgt |
| 119*lacY*-mid-R | gtgttttttaaatagtacatggttaattcctcctgttacg |
| 119*lacY*-down-F | cgtaacaggaggaattaaccatgtactatttaaaaaacac |
| *lacY*-down-R | tgattattgatggtgaacat |
| GWBC-up-F | tgtttctcaattcgggcgat |
| 119GWBC-up-R | ggaggatattctcgagactatggttttccttatattcaga |
| 119GWBC-mid-F | tagtctcgagaatatcctcctttaccgTTCGTATAATGTATG |
| 119GWBC-mid-R | ggttaattcctcctgttacg |
| 119GWBC-down-F | cgtaacaggaggaattaaccatgtcaaaagtcgctctcat |
| GWBC-down-R | tgatccagtaggcgtacagt |
| ***gapA* regulation** |  |
| *gapA*-up-F | cagttaccgcaacgaatttc |
| 119*gapA* -up-R | ggaggatattctcgagactactgactcgcctcactcttcc |
| 119*gapA*-mid-F | tagtctcgagaatatcctcctttaccgTTCGTATAATGTATG |
| 119*gapA*-mid-R | ggttaattcctcctgttacg |
| 119*gapA*-down-F | cgtaacaggaggaattaaccatgactatcaaagtaggtatcaa |
| *gapA-*down-R | gatgccgaagttatcgttga |
| 119-AGG-*gapA*-down-F | cgtaacaggaggaattaaccatgaggactatcaaagtaggtatcaa |
| 119-AGGAGG-*gapA*-down-F | cgtaacaggaggaattaaccatgaggaggactatcaaagtaggtatcaa |
| 119-AGGAGGAGG-*gapA*-down-F | cgtaacaggaggaattaaccatgaggaggaggactatcaaagtaggtatcaa |
| 119-AGGAGGAGGAGG-*gapA-*down-F | cgtaacaggaggaattaaccatgaggAGGaggaggactatcaaagtaggtatcaa |

**Fig. S1** Three routes of fructose intake in *E. coli* ^[1]^


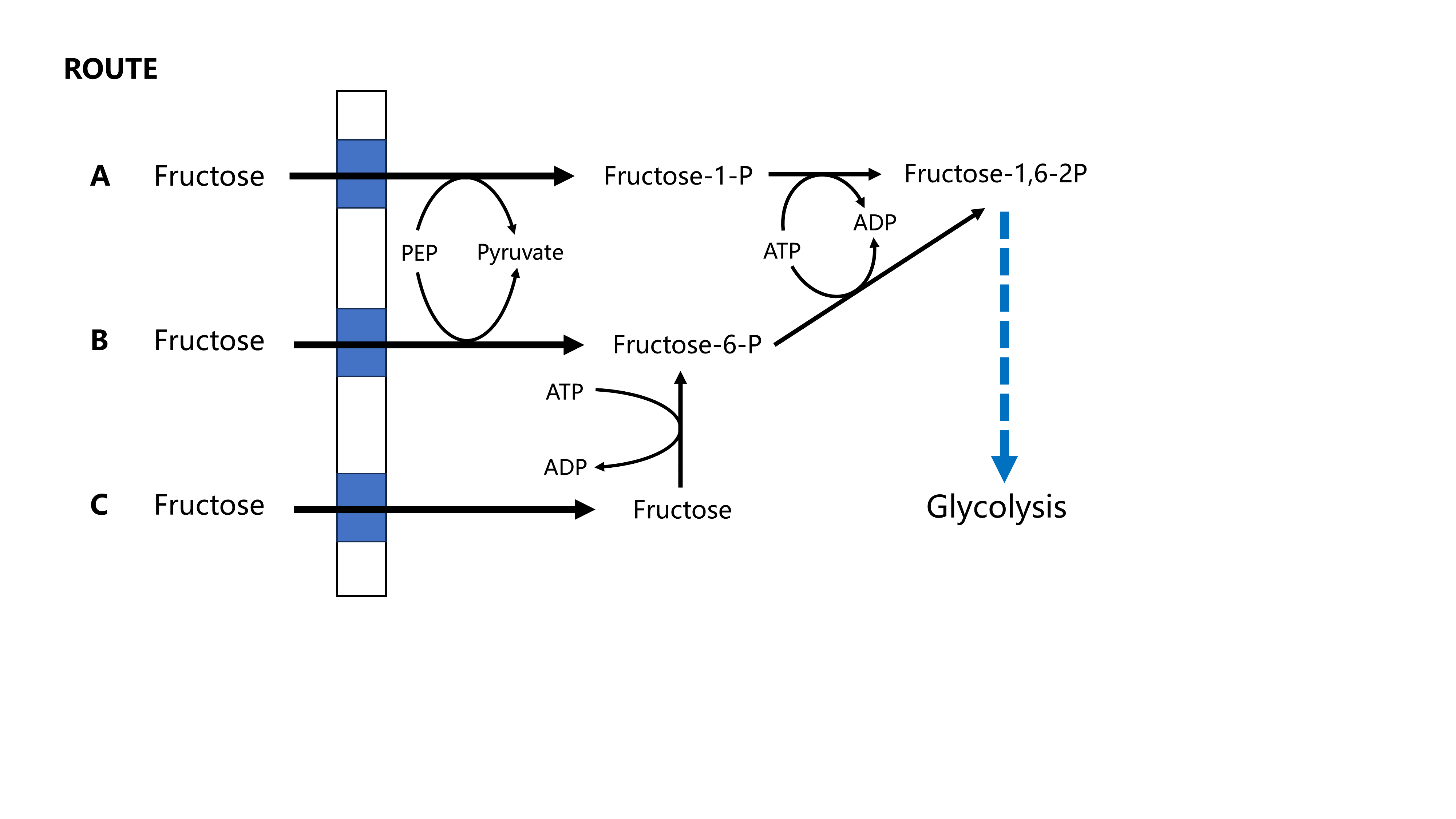


**Fig. S2** The expressions of 2’-FL biosynthesis pathway enzymes (ManC, approximately 53 kDa; ManB, approximately 50 kDa; Gmd, approximately 42 kDa; WcaG, approximately 36 kDa; FucT, approximately 35 kDa) in different chassis strains. The “↑” and “↓” respectively correspond to supernatant and sediment.


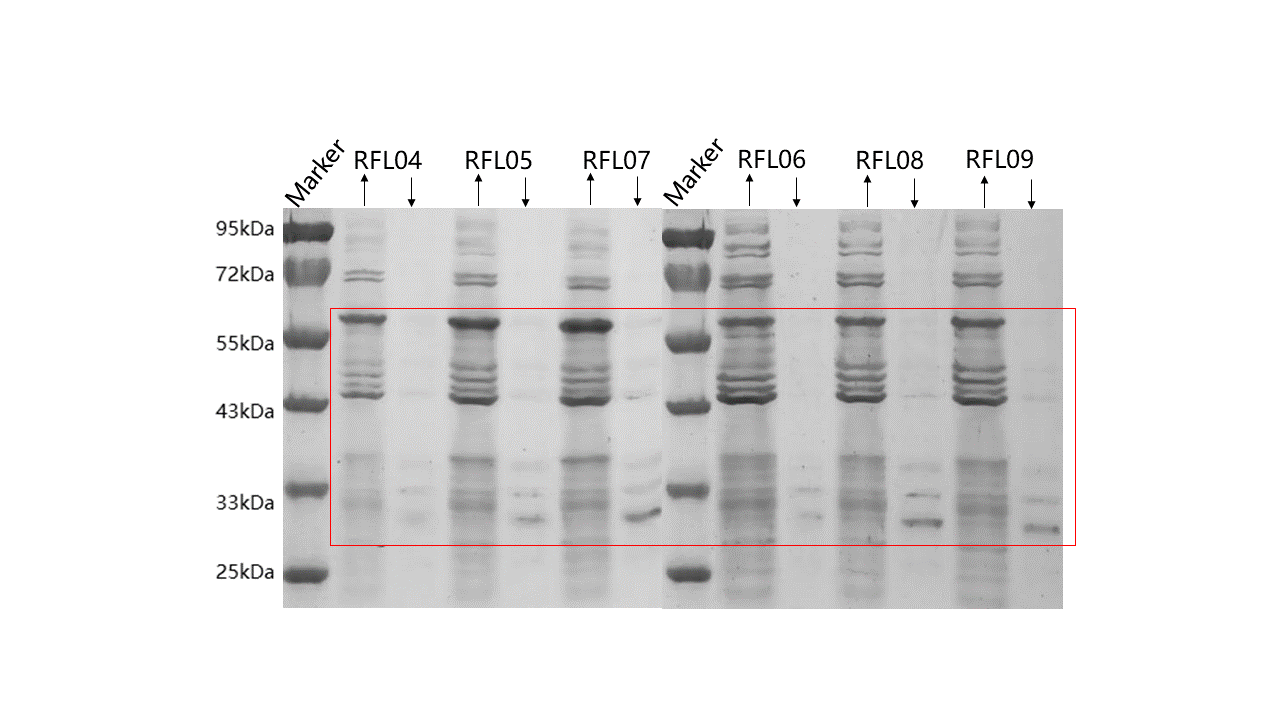


**Fig. S3** The scaled-up bioconversion of 2’-FL by strain RFL38. **a** The expression of key enzymes of 2’-FL biosynthesis pathway. **b** Production of 2’-FL by strains RFL38 cultivated in 1-L fermenter. The bioconversion was in a 1-L fermenter containing biomass (OD_600_ = 20), 100 mM lactose, 2 mM MgSO_4_, and 1 × M9 salts buffer. Fructose was added in batches; 70 mM fructose was added at 0 h, 100 mM fructose was added at 9.5 h, and 25 mM fructose was added at 24 h.


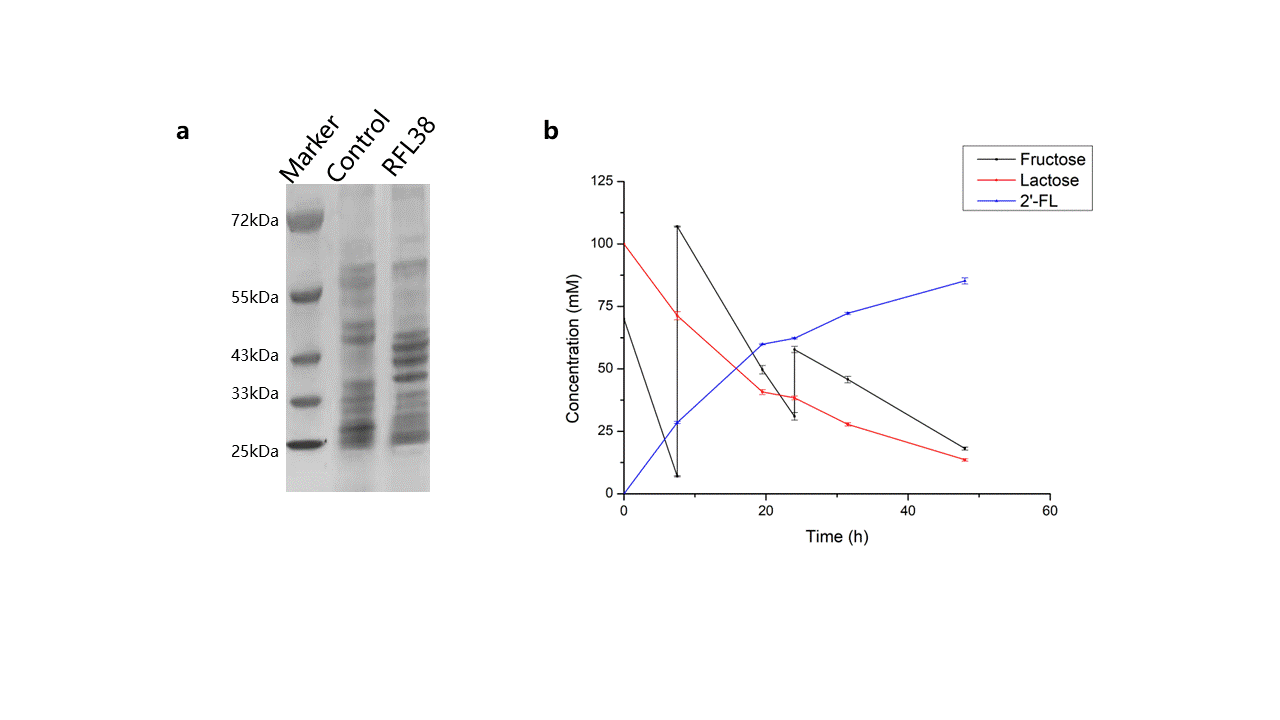


**Fig. S4** HPLC chromatograms of 2’-FL, glucose, fructose, and lactose. The x-axis shows retention time and the y-axis the refractive index detector (RID) signal. The retention times of 2’-FL, fructose and lactose were 8.421 min, 9.109 min, and 11.715 min respectively.


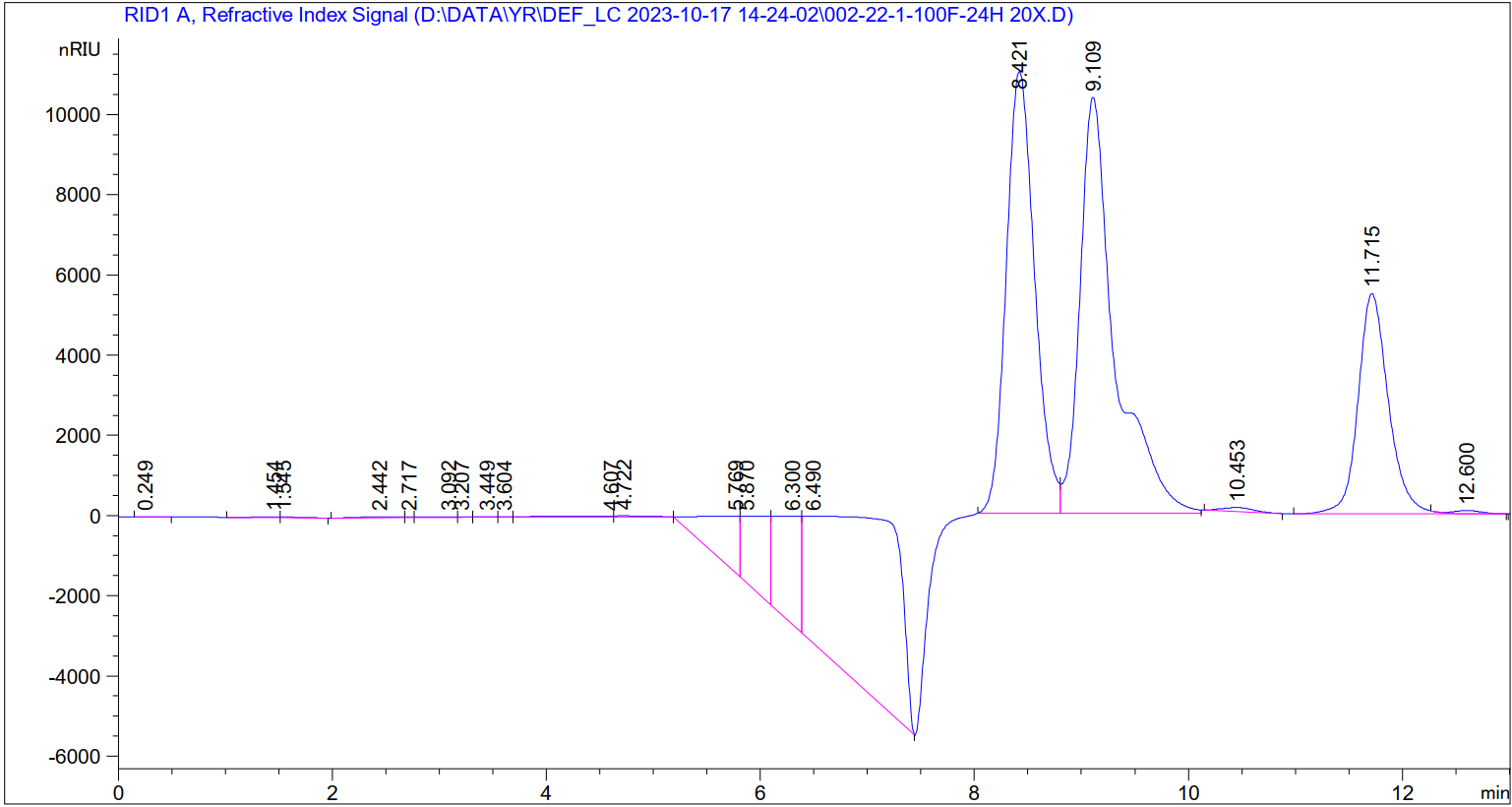


Reference

1. Kornberg HL: **Routes for fructose utilization by Escherichia coli.** *J Mol Microbiol Biotechnol* 2001, **3:**355-359.
